# Trial encoding algorithms ensemble

**DOI:** 10.1186/2193-1801-2-316

**Published:** 2013-07-15

**Authors:** Lipin Bill Cheng, Ren Jye Yeh

**Affiliations:** Cavalry Storage, Inc, 4251 E Brickell St, Ontario, CA 91761 USA

**Keywords:** Symmetric encryption, Digital signature, Hash, Error correcting code, Bit compression

## Abstract

This paper proposes trial algorithms for some basic components in cryptography and lossless bit compression. The symmetric encryption is accomplished by mixing up randomizations and scrambling with hashing of the key playing an essential role. The digital signature is adapted from the Hill cipher with the verification key matrices incorporating un-invertible parts to hide the signature matrix. The hash is a straight running summation (addition chain) of data bytes plus some randomization. One simplified version can be burst error correcting code. The lossless bit compressor is the Shannon-Fano coding that is less optimal than the later Huffman and Arithmetic coding, but can be conveniently implemented without the use of a tree structure and improvable with bytes concatenation.

## Introduction

This paper proposes trial encoding algorithms for some common primitives in cryptography and lossless bit compression. The symmetric encryption is slightly faster than some common block ciphers. It consist of randomization + scrambling (permutation) + randomization. The randomization keys (seeds) are the hashes of the key and the scrambling key is the key itself. The IV is hidden (a costly practice, thus optional) to compensate for possible unevenness in the IV/initial generator state relation. The digital signature is an adaptation of the Hill cipher by hiding the signature key matrix with un-invertible verification matrices and turned around into a signature scheme. They are substantially faster than some common schemes. Key pairs can be derived straight from any user specified code for example, user password. The hash has similar speed to some existing ones. Its collision resistance comes from a substantial mixture of non-linear functions to thwart differential cryptanalysis. One non-cryptographic version can be burst error correcting code. The Shannon-Fano bit compressor is not always optimal like Huffman coding, but simple enough that a medium array may be used as the data structure in place of a binary tree. It can also be complemented with bytes concatenation and Rice coding for better compression. The cryptography part generally follows the guideline of essential cryptographic practices as illustrated in the article “An Overview of Cryptography” (Kessler 2013). Miscellaneous details of relevance including source codes are in “An Encoding Kit”(Lipin Bill 2013).

The test/coding platform for all benchmarks in this document is shown in Table 1.

**Table 1:** **Test/coding platform for all benchmarks**

Hardware	processor	OS	IDE	Framework
Acer laptop	AMD E-350 1.60 GHZ 4.0 GB RAM	Windows 7 64-bit	VISUAL WEB DEVELOpER 2010 EXpRESS	ASp.net C#

## Symmetric encryption

First generate a random number as the IV (initialization vector). IV is encrypted because among other issues, it is an input parameter which is partially under the control of the adversary (Erik 2007). Data is randomized (stream cipher like), followed by scrambling (block cipher like), and ending with another round of randomization. For convenience, this is named RSC (randomization scrambling cipher).

### The algorithm

The random key stream generation is based on the cyclic piling up of modular addition, XOR, AND, increment, and byte permutation of the key (seed).

This generator is compared with the secure random generator from C# system call. No differences have been detected in some statistical randomness tests (frequency, gap, runs, poker and serial) (Lipin Bill 2013; Lott 2005). It also passed all NIST randomness tests (Andrew et al. 2010), as shown in Table 2: bit sequence length = 100,000 (except 1,000,000 for Universal Statistical test), pass decision = p-value > 0.01 (significance level).

**Table 2:** **NIST randomness tests of key stream generator**

Randomness test	p-value	Result
Approximate entropy	0.526869	Success
Block frequency	0.431942	Success
Cumulative sums	0.609751	Success
FFT	0.450552	Success
Frequency	0.775947	Success
Linear complexity	0.450531	Success
Longest runs	0.223268	Success
Non-overlapping template	0.438379	Success
Overlapping template	0.263724	Success
Random excursions	0.350888	Success
Random excursions variant	0.221961	Success
Rank	0.461483	Success
Runs	0.203627	Success
Serial	0.641047	Success
Universal statistical	0.170547	Success

The bit scrambling (permutation) is a bit by bit swap with a bit decided by the key.

putting it altogether:

(6 bytes) Key is K = (K1 K2 K3 K4 K5 K6). Data is D = D1 D2 D3 …

Generate random R = (R1 R2 R3 R4 R5 R6) as the IV (initialization vector).

Get H = hash of K. Any efficient hash can be used, but better a dedicated one (Erik 2007).

Set IV = R ^ H.

Scramble IV with K as the scrambling key.

Get H = hash of H.

Set IV = IV + H.

Compute K = K ^ R.

Get H = hash of K.

Feed H as the seed to the pseudorandom generator to get a random R.

Now set D1 = D1 ^ R.

Continue with the pseudorandom generator to get another random R.

Now set D2 = D2 ^ R.

Repeat this to the last data byte.

Scramble the data either as a whole or block by block for better performance with K as the scrambling key. When the data bit index reaches 6 x 4 = 24 bytes = 192 bits, bit scrambling is switched to byte scrambling since 24! > 2 ^ 48, because bit scrambling is expensive.

Get H = hash of H.

Do another round of randomization with the same pseudorandom generator and H as the seed, but set D = D + R.

Concatenate IV and D to wrap up encryption.

Decryption is the natural reverse of encryption.

### Security aspect

The initial inner state of the key stream generator is the hash of K + IV, and should be indistinguishable from random (Erik 2007). Since theoretically hash does not meet that criterion, IV is randomly generated to begin with, also encryption of IV is intended to mitigate the potential problem assuming that encrypted IV is no less random than plain IV. Evidently the goal is to break any exploitable predictability of the initial state from the encrypted IV. Statistical tests were thus conducted to help determining whether this is effective. For a given E1/E2 differential, no statistical deviation from random has been found in the H1/H2 differentials, where E is an encrypted IV and H is the corresponding initial state (Lipin Bill 2013). These differentials also passed all NIST randomness tests (Andrew et al. 2010). As a rule of thumb, the key/IV size complexity should be 4 times the length of data to be encrypted. The size of the inner state is 2 times the key size, but can be increased (at the expense of performance), as there are indications in the literature that 4 times is safer. The IV encryption is made of a XOR and a modular addition with a bit scrambling sandwiched in between. The bit scrambling results in brute force complexity of the order of n!/((n/2)! (n/2)!) ≈ 2 ^n^, where n is the key size in bits. However, IV encryption is only optional.

Data encryption is similar to IV encryption: 1 round of XOR key stream randomization and 1 round of modular addition key stream randomization with 1 round of scrambling sandwiched in between. Confusion obviously comes from the combined actions of all 3 rounds while diffusion is accomplished by the scrambling round which is optional, as OTp and its weaker versions, stream ciphers are provably/acceptably secure with confusion only. The brute force complexity is similar to that of IV encryption, with the effective key size being the lesser of data and key. Key space of the randomizations are made of 2 hashes of the key. The scrambling key is the key itself. In addition, because the IV is not a mere additive to 1st block of plaintext, it has to be factored into the linear approximation of a known plaintext encryption. In other words, linear cryptanalysis will have to start with IV cracking (except for man-in-the-middle attack where the attacker could intercept the communications and change the IV on the fly (Frederic 2004)), followed by modification of the key with the IV addition and the subsequent hashing. Another issue is that the random IV is not a nonce, its birthday problem (if IV is deemed as not long enough) is thus rectified with the removal of XOR malleability by mixing up randomizations and scrambling.

As input has no bearing on key stream generation, plaintext input differential cryptanalysis is not applicable to RSC. The main issue here is with the key differential. Since a key stream is primarily made of modular addition and XOR, obviously these relations between a pair of keys K1 and K2 will be carried over to their respective key streams, that is, had it not been for hashing and IV. The seeds to the key streams are H(K1 + IV1) for one and H(K2 + IV2) for the other. The K1/K2 differential is not preserved in H1/H2. So such attacks cannot be trivially effective. Similarly, some stream ciphers were subject to plaintext IV attacks on the key, for example, it was shown that two IV’s with some given difference may produce the same key stream (Eli & Orr 2007). However, because the IV1/IV2 relation does not go to H1/H2, this cannot be effectively applied to RSC either, although more study is still needed on whether the initial generator state is random enough relative to the (encrypted) IV.

### performance aspect

RSC benchmark is shown in Table 3.The performance is basically independent of the key size and slightly faster than some of the well-known block ciphers. This is conceivably because RSC does mostly byte scrambling (if data is over 10 times bigger than the key) rather than bit scrambling (permutation). But the initial stage is slowed down by random IV generation, IV encryption, key hashing and bit scrambling.

**Table 3:** **RSC benchmark relative to common ciphers - data size: 1,000,000 bytes**

Cipher	Key	Encryption time	Decryption time
AES	256 bits	80 ms	90 ms
Rijndael	256 bits	150 ms	150 ms
3DES	192 bits	120 ms	120 ms
RC2	128 bits	70 ms	400 ms
Blowfish	64 bits	500 ms	250 ms
RSC	256 bits	40 ms	40 ms

### Symmetric encryption summary

As is well known, this is the black/fine art of striking a balance between the conflicting demands of security and performance. This is true of both the key stream generation and scrambling. The general concept is to produce just enough randomness with minimum operations to ward off some known attacks. But it comes with a costly initial stage where IV is hidden (contrary to common practice, although optional).

## Digital Signature

This algorithm is an adaptation of the Hill Cipher (Murray 1999; John & Matthew 2010; Aldar 2009), thus named HDS (Hill digital signature) for convenience.

### The Algorithm

**Step 1.** Generate 2 x 2 random invertible matrices A1 and A2 along with *A1*^-1^ and *A2*^-1^. Now choose constant un-invertible matrices C1 and C2:

$$C1\;=\left[\begin{array}{cc} 1 & 1\\ 1 & 1 \end{array}\right]\;\text{and}\;C2\;=\;\left[\begin{array}{cc} 1 & -1\\ -1 & 1 \end{array}\right]$$

where C1 + C2 = 2 x I and I is identity matrix.

**Step 2.** Verification key is V1 = C1 x A1 and V2 = C2 x A2. Signature key is S1 = *A1*^-1^ and S2 = *A2*^-1^.

**Step 3.** To sign D, generate random numbers R1 and R2, such that C1 x R1 = 0 and C2 x R2 = 0. Then compute E1 = S1 x (R1 + D) and E2 = S2 x (R2 + D). R1 and R2 are added to prevent plaintext attacks upon the signature key.

**Step 4.** To extract/verify D, compute (V1 × E1 + V2 × E2)/2.

$$\begin{array}{l} \left(V1\times E1+V2\times E2\right)/2\\ =(C1\times A1\times A1{\;}^{-1}\times \left(R1+D\right)+C2\times A2\times A2^{-1}\times \\ \;(R2+D))/2\\ =\left(C1\times R1+C2\times R2+\left(C1+C2\right)\times D\right)/2\\ =\left(2\times D\right)/2=D \end{array}$$

With half the matrix missing, the signature key is quite clearly not derivable from the verification key. Hence presumably the only viable way to fake a signature is to attack the verification formula. This amounts to solving the linear equations V1 x E1 = D1 and V2 x E2 = D2, where D1 = {(d1 + d2), (d1 + d2)} and D2 = {(d1 – d2), – (d1 – d2)}. But this cannot be done because V1 and V2 are un-invertible matrices. The verification key size is the matrix size which can be for example, 2 x 2 x 32 bytes = 128 bytes. The signature faking complexity is assumed to be 1 / 2 of the missing matrix size, or 1 / 4 of the matrix size = 128 / 4 = 32 bytes. Thus the ratio of verification key size to effective key size is 128 / 32 = 4 times. The data size expansion after signature is typically about 4 times.

### performance aspect

AHC benchmark is shown in Table 4, indicating faster speed than the other algorithms tested. Its operation time is proportional to the key size.

**Table 4:** **HDS benchmark comparisons - Data size: 80 bytes**

Signature	Key	Effective key	Key gen-eration time	Signature time	Verification time
RSA	1024 bits	80 bits	200 ms	6 ms	5 ms
DSA	?	?	300 ms	5 ms	3 ms
ECDSA	?	?	50 ms	13 ms	22 ms
HDS	1064 bits	256 bits	20 ms	0.3 ms	0.3 ms

### Digital Signature summary

Matrix pairs are generated by a pseudorandom generator with a user specified seed (thus keys can be generated on the fly). Then with un-invertible matrices as part of the verification key, Hill cipher is adapted into a digital signature scheme. However, it has an overhead in bigger key size and data size expansion after signature.

## Hash functions

This hash tallies running modular summations (addition chain) of data bytes. Another round of running XOR’s is tacked on, mixed with more non-linear functions, and named matter-of-factly RSH (running summation hash) for convenience.

### The algorithm



Optionally, salting may be added to prevent pre-calculated rainbow table attacks.

### Security aspect

In essence, the pre-image resistance is to match the hash size. The collision resistance is to match the inherent birthday problem’s one half of the hash size. pre-image resistance alludes to hash being one way, given a specific message M, it’s computationally infeasible to derive M or any of its peers (collisions, if M is bigger than H) from H(M). Collision resistance is the same definition with the word “specific” changed to “arbitrary”. From a practical point of view, the only difference between the two is that birthday attacks can be applied to finding collisions, but not to pre-images.

RSH’s collision resistance relies on the assumed hardness to solve a system of non-linear equations. If we replace all modular additions by XOR’s in a hash function operating on n–bit words (bytes) and originally containing r modular additions, we get probability(3/4) ^r (n – 1)^ that the output is the same as in the original hash function (Nieke 2011). So linearization cannot help much with cryptanalysis, as this is not much different from the brute force complexity in order of magnitude.

However, all hashes are subject to differential cryptanalysis somewhat like all symmetric ciphers except OTp are subject to linear cryptanalysis. While linear cryptanalysis cannot be applied to hashes (unless the non-linear part of hashing is really weak) because there is no such thing as a “partial” collision, there is no escape from exploitation of uneven differentials among hash inputs and outputs. Assuming for example, that the hash is strictly linear with respect to XOR (just for convenience of discussion, not realistic because pre-images can be directly solved and all collisions found), then for a given input A, any input B such that H(A ^ B) = 0 would be a collision. The implication is that however poor a linear approximation may be, a differential input pair would give a related output pair (not entirely random). As a realistic example, if a hash is made of modular addition and XOR, then it can be attacked with combined differentials of modular addition and XOR (Nieke 2011).

RSH’s approach is to “maximize” non-linearity without losing performance too much. The non-linearity comes from mixing up modular addition and XOR, modular multiplication of the running summation with the running index of looping in postproc(), bit permutation in postpermute() and some AND’s at the end. Therefore, combined differentials attacks have to include modular multiplication, bit permutation and AND.

The optimum birthday attack resistance is n/2 bits, where n is the hash size. This is equivalent to the hash output being even – random input leads to random output, so does perfectly ordered input. No statistical deviation from random has been found in tests conducted for this (Lipin Bill 2013). These outputs also passed all NIST randomness tests (Andrew et al. 2010).

### performance aspect

RSH benchmark is shown in Table 5. Its speed is basically independent of the hash size and quite similar to some of the hashes currently available except for shorter hashes.

**Table 5:** **RSH benchmark relative to common hashes - data size: 10,000,000 bytes**

Hash	Hash size	Compute time
MD5	128 bits	50 ms
RIpEMD 160	160 bits	240 ms
SH1	160 bits	80 ms
SH256	256 bits	240 ms
SH512	512 bits	180 ms
RSH	512 bits	180 ms

### Non-cryptographic hash – error correcting code

The 1 pass only version is tailor made for data error detection and correction by solving the quasi-linear equations set of erroneous data bytes (if they are contiguous) out of the hash. If there is a unique solution, then the error can be corrected.

A few extra bytes of encodings are attached to the hash in order to get a unique error correcting because the equations to be solved are quasi-linear (overflow discarded). If the (recalculated) hash is changed upon data reception indicating errors, then it is possible to restore the corrupted bytes from the hash and the attached bytes.

### Summary of hash functions

2 rounds of running summations and/or XOR’s form the backbone of hashing. With additional non-linear functions packed into the hashing, methodically cracking the collision resistance may be no faster than brute force birthday attacks. The 1 pass only version can be used for burst data error detection and correction.

## Bit compressor

This is a simpler precursor of Huffman coding. In essence, it is just a bit representation that reflects the actual probability of each number in the data. This is in contrast to the “regular” bits that assume all possible numbers are equally likely in the data. For example, byte 21 is equal to or less than 127, therefore the 1st bit is 0, then it is compared against 63 and so on, thus arriving at 00010101. But for a typical piece of text data, some bytes would be more frequent than the others. Therefore, the 1st comparison would be with something less than 127, the next would be less than 63, and so on. Thus, it presumably would result in shorter bits along the same line of the later and more optimal Huffman coding, as proposed by Shannon and Fano, and therefore named SFC (Shannon-Fano coding) for convenience.

### The algorithm

As described above, if the data is random, then the medium byte array would be 127, 63, 31, 15, 7, 3, 1, 0, 1, 2, 3, 5, 4, 5, 6, 7, 11, … But for text data, it could be something like 10, 4, 2, 1, 0, 0, 1, 2, 3, 3, 4, 7, … These are derived from the frequencies (same as Huffman coding) of each data byte. It is sent along with the compressed data. Being an array, it is easier to handle than the binary tree in Huffman coding.

As a simple illustration, assuming there are 6 different bytes in the data with the following counts in non-ascending order:

Cnt[] of {15, 76, 59, 123, 68, 154} = {40, 15, 10, 5, 2, 1}

Map {15, 76, 59, 123, 68, 154} to N[] = {0, 1, 2, 3, 4, 5}

Now construct the medium array M[] for N[]:

The 1st element in M[] is the medium of N[]: add up 40 + 15 + 10 + 5 + 2 + 1 = 73, and since 40 >= 73/2, so M[0] = 0.

M[1] is the medium of all elements in N[] that are <= M[0]. Since there is only one such element, M[1] = M[0] = 0. M[1] is also an “end” element (the only one).

M[2] is the medium of all elements in N[] that are > M[0]: 15 + 10 >= (15 + 10 + 5 + 2 + 1)/2, so M[2] can be 2, but 1 is closer to medium, so M[2] = 1.

M[3] is the medium of all elements in N[] that are > M[0] and <= M[2]. Since there is only one such element, M[3] = M[2] = 1. M[3] is also an “end” element.

…, thus arriving at the medium array, M[] = {0, 0, 1, 1, 2, 2, 3, 3, 4, 5}. Some rearrangement is made to this array to shorten it before eventually attaching it to the compressed data. But this is not critical, as this array is typically quite short compared with the data. There is also a Boolean array associated with the medium array, E[] = {0, 1, 0, 1, 0, 1, 0, 1, 0, 1}, indicating if the element is at the end (of a search).

0 Byte 0 mapped to bits:

<= M[0], followed by 0 <= M[1] and also E[1] = 1 (reaching end), so Bits[0] = 0.

Byte 1 mapped to bits:

1 > M[0], bypass M[1] to get to the next element greater than M[0], so that’s M[2], 1 <= M[2], followed by 1 <= M[3] and also E[3] = 1, so Bits[1] = 10.

Byte 2 mapped to bits:

2 > M[0], bypass M[1] to get to the next element greater than M[0], so that’s M[2], 2 > M[2], bypass M[3] to get to the next element greater than M[2], so that’s M[4], 2 <= M[4], followed by 2 <= M[5] and also E[5] = 1, so Bits[2] = 110.

…, thus Bits[] of {0, 1, 2, 3, 4, 5} =

0

1 0

1 1 0

1 1 1 0

1 1 1 1 0

1 1 1 1 1

So data compression just maps bytes 0, 1, 2, … to the corresponding Bits[].

Decompression reverses this process, mapping the bits back to 0, 1, 2, …, then back to 15, 76, 59, 123, 68, 154.

Depending on the text size, some common words may show up quite often. So concatenation of data bytes into bigger units may pay off in achieving better compression. For better performance, the principle of Rice coding is borrowed here. That is, the compression is done not on the data itself, but on the number of bits in each data unit. The (transformed) data stripped of the top bit (must be 1) is sent as it is. Decompression extracts the recorded number of bits back into each unit. Thus compression is primarily data mapping, with bit conversion playing a lesser role. This (recursive) concatenation scheme is somewhat like dictionary coding, such as LZW, in that it can also be applied to the likes of GIF, pNG and clip arts.

### performance

SFC benchmark is shown in Table 6. Compression and speed are near identical with Huffman coding for common texts, unless data frequency distribution is significantly “undividable”.

**Table 6:** **SHC benchmark comparisons - Text size: 1,083,745 bytes**

Compressor	Compression	Compression time	Decompression time
Huffman coding	744,298 bytes	200 ms	?
Arithmetic coding	733,356 bytes	450 ms	700 ms
SFC	740,441 bytes	200 ms	130 ms
SFC concatenated	650,225 bytes	280 ms	180 ms

### Summary of Bit compressor

This calculates the frequency of each data byte just like Huffman coding. But instead of a binary tree as in Huffman coding, an easier to handle medium array is constructed. The downside of this is that the bit conversion is not always optimal. This happens when a medium deviates significantly from evenly dividing a set of data. Conversion of a data byte to (shorter) bits is done by comparing it with the medium array which is then replaced by a mapping table for speed. Where appropriate, data bytes may be concatenated into bigger units for better compression while largely maintaining its speed.

## Conclusions

Some trial encoding algorithms were proposed. Their implementation is quite simple relative to some comparable encoders. performance comparisons with some well-known similar algorithms were conducted. The results show better or no worse performance: the symmetric cipher has a slight edge over some common block ciphers, the digital signature can run considerably faster than some common schemes, the hash and the bit compressor are near the same with some of the most common. To sum up the approaches: optional encryption of the IV at the outset, followed by 2 rounds of data randomization with 1 round of data scrambling sandwiched in between in the symmetric cipher, adaptation of Hill cipher by use of un-invertible matrices as part of the verification keys in the signature scheme, chaining of addition/XOR/multiplication, followed by bit permutation and ending with some AND’s in the hash. The bit compressor is slightly different from Huffman coding that uses an array rather than a binary tree as the data structure, with an advantage and a disadvantage. Compression may be enhanced through bytes concatenation and Rice coding. A non-cryptographic version of the hash is for burst error correction.

## A. Appendix

### A.1 CSpRNG proposal

All encryptions and signatures require randomization. The C# CSpRNG (cryptographically secure pseudorandom number generator) can be used for this purpose. Alternatively, the following generator is proposed for better performance.

This is the 2 rounds of randomizations in RSC wrapped into one. Like an individual round, it also passed all NIST randomness tests (Andrew et al. 2010).

To function as a stream cipher, let key1 be the IV randomized secret key and key2 be the hash of key1.

### A.2 RSC with nonce

Rather than random IV’s, a variation of RSC can use IV that is nonce (non-repeating number), the simplest of which is a plain sequence, 0, 1, 2, 3, … Nonce ensures each encrypted message can only be valid once without incorporating a time stamp or unique ID in the plain message, thus cannot be re-used over the internet by hijackers.

IV is transmitted in the open (no encryption).

Scrambling key = SK = pRF(K + IV), where pRF is a pseudorandom function.

1st randomization key = RK1 = pRF(SK).

2nd randomization key = RK2 = pRF(RK1).

The pRF here should be computationally indistinguishable from random, leading to a costly initial stage in the encryption. But depending on the strength of the underlying key stream generator, this requirement can be relaxed for better efficiency (Erik 2007). In lieu of a dedicated pRF that is yet to be found in the literature, the following series of RSH based key generators is proposed. The idea is to list them in increasing complexity (thus decreasing efficiency), with the optimum choice being the one with best efficiency, yet enough security for a particular situation. Given that the in-distinguishability from random is a generally sufficient, but not always necessary condition for a viable pRF, all proposals are therefore tested (for plain sequence input) with the NIST randomness test suite, as a general reference to their relative strengths.

keyHash_1() (2nd round removed) and keyHash_2() (modular multiplications removed) are simplified versions of postproc(). They are the only ones failing the NIST randomness tests, but nonetheless likely sufficient for the initial generator state setup given random IVs and possibly for ordered nonce IVs too, although more study is needed for that. So it appears that in general, keyHash_3() is the best tradeoff between security and efficiency. In fact, it is the one used in RSC. On the other hand, keyHash_4() and keyHash_5() may be “overkills” in typical situations, as postproc() alone passed all NIST randomness tests for plain sequence input.

### A.3 RSC scrambling

This is to break any exploitable correlation between input and output (diffusion). For testing purposes, the input is kept simple, 10 random bytes, followed by 0’s. Bit scrambling is shown, so is byte scrambling which is sufficient to accomplish diffusion if input size is substantially larger than key size. Actual scrambling is mixed.

Input:

39 237 13 203 234 75 123 250 185 147 0 0 0 0 0 0 0 0 0 0 0 0 0 0 0 0 0 0 0 0 0 0 0 0 0 0 0 0 0 0 0 0 0 0 0 0 0 0 0 0 0 0 0 0 0 0 0 0 0 0 0 0 0 0 0 0 0 0 0 0 0 0 0 0 0 0 0 0 0 0 0 0 0 0 0 0 0 0 0 0 0 0 0 0 0 0 0 0 0 0

Bit scrambling output:

132 161 36 2 145 129 136 0 4 224 96 0 129 192 160 16 0 0 64 0 1 64 160 0 0 0 96 0 0 0 128 16 0 0 64 16 0 0 0 0 0 0 192 16 0 0 0 0 0 0 64 0 0 0 0 0 0 0 64 0 0 0 0 0 0 0 0 0 0 0 0 0 0 0 64 0 0 0 64 0 0 0 0 0 0 0 0 0 8 0 0 0 8 0 0 0 0 0 0 0

Byte scrambling output:

0 13 0 0 0 0 0 0 0 0 0 0 0 0 0 39 147 0 0 0 0 0 0 0 0 0 0 0 0 0 0 0 203 0 0 0 0 0 0 0 0 0 0 75 0 123 0 0 0 0 0 0 0 0 0 0 0 0 0 0 0 0 0 0 0 185 0 0 0 0 0 0 0 0 0 0 0 0 0 0 0 250 234 0 0 0 0 0 0 0 0 0 0 237 0 0 0 0 0 0

RSC (mixed) scrambling output:

26 16 67 9 6 0 0 0 10 32 1 0 7 0 0 0 0 0 0 0 15 32 4 0 0 6 0 0 0 6 0 0 0 0 0 0 8 0 1 0 8 0 0 0 0 0 0 0 0 0 0 8 0 32 0 0 0 0 0 0 0 32 0 0 0 0 0 0 4 0 0 0 4 0 0 0 0 32 0 0 0 0 0 0 0 0 0 0 0 0 0 0 0 0 0 0 0 0 0 0 0 0 0 0 0 0 0 0 0 0 0 0 0 0 0 0 0 0 0 0 0 0 0 0 0 0 0 0 0 0 0 0 0 0 0 0 0 0 0 0 0 0 0 0 0 0 0 0 0 0 0 0 0 8 0 0 0 0 0 0 0 4 2 0 0 0 0 0 0 0 0 0 0 0 4 0 0 0 0 32 0 0 0 0 0 7 4 0 0 0 32 0 0 8 0 0 0 0 0 0

### A.4 RSC linear cryptanalysis

For simplicity, only the 1st round of randomization is taken into consideration here (just for illustration, as 1 round of randomization can easily be cracked with divide and conquer, rather than the pretentious method below):

C1 = p1 ^ S1, C2 = p2 ^ S2, …, Cn = pn ^ Sn, where C is a cipher byte, p is a plain byte, S is a key stream element and n is the key size in bytes. From the key stream algorithm, S1 = (K1 ^ Kn) + Kn, S2 = (K2 ^ S1) + S1, S3 = (K3 ^ S2) + S2, …, therefore the total number of modular additions in these equations is effectively n. If we replace all the modular additions with XOR, then the accuracy of the resulting linear approximation is (3/4)^n (8 – 1)^(Nieke 2011), or (3/4) ^112^_≈_ (1/2) ^50^, if n = 16 bytes. An effective attack thus requires the order of 2 ^50^ known plaintexts, hence is generally impractical.

In addition, there is another round of randomization and the effect of one bit of IV change leading to an entirely different randomization key, H(K + IV).

### A.5 Matrix pair generator

HDS uses a fast matrix pair generator that creates a matrix as (I + Δ1) x (I + Δ2) x (I + Δ3) x … where I is the identity matrix, Δ is a random matrix with all 0 except one non-diagonal element is 1 or –1. All the random numbers used in the matrix generation can be from a pseudorandom generator with a user specified seed. The inverse is by virtue of the property (**A**_1_**A**_2_⋯**A**_*k–1*_**A**_*k*_)^–1^ = **A**_*k*_^–1^**A**_*k–1*_^–1^⋯**A**_2_^–1^**A**_1_^–1^. The inverse of each component is the same except that the non-zero non-diagonal element is negated. Besides keeping the matrix simple, it is faster than inverting an existing matrix when matrix dimension reaches 20 × 20.

### A.6 HDS Signature Faking

Faking is obtained by solving the linear equation, V x E = D, or (C x A) x E = D. If the matrix dimension is 2 x 2, this reduces to a1 x e1 + a2 x e2 = k1, where k1 = d1 + d2. The expected fake resistance is 1 / 4 of the matrix size. Test results indicate similar order of magnitude between the actual and expected fake resistance, as shown in Table 7.

**Table 7:** HDS signature faking benchmark – expected fake resistance = sqrt(a1 x a2)

Test	a1	a2	e1	e2	k1	Resist’ce	Expected
1	-2797146	-18277625	-757510776	115927001	2014607329	7270925	7150188
2	-56824	-196635	2140009268	-618418855	-1095091907	97915	105705
3	16106711	-3810926	-1261293830	-5330803243	1473739888	3574125	7834634
4	-2050051	-5798512	-1302033155	460330610	1805388585	878573	3447788
5	-2314423	12407679	86461160	16127823	1153442137	36454461	5358789
6	3760308	25672051	1519003560	-222495726	-386457546	3850777	9825213

### A.7 RSH evenness

Each input instance is 64 bytes. Input = {0, 0, 0, …}, {1, 0, 0, …}, …, {255, 0, 0, …}, {0, 1, 0, …}, {1, 1, 0, …}, …, {255, 1, 0, …}, {0, 2, 0, …}, {1, 2, 0, …}, … NIST randomness test of the output is shown in Table 8: bit sequence length = 100,000 (except 1,000,000 for Universal Statistical test), pass decision = p-value > 0.01 (significance level). The output is quite even despite unevenness of input. This raises the possibility of customizing RSH into a pRF for the IV setup in a cipher.

**Table 8:** **NIST randomness tests of RSH for ordered input**

Randomness test	p-value	Result
Approximate entropy	0.099491	Success
Block frequency	0.496425	Success
Cumulative sums	0.391556	Success
FFT	0.296168	Success
Frequency	0.330066	Success
Linear complexity	0.662034	Success
Longest runs	0.553321	Success
Non-overlapping template	0.436819	Success
Overlapping template	0.602813	Success
Random excursions	0.589878	Success
Random excursions variant	0.121938	Success
Rank	0.339569	Success
Runs	0.324969	Success
Serial	0.410914	Success
Universal statistical	0.571653	Success

### A.8 RSH divide and conquer

One potentially viable approach to crack the collision resistance is to attack the 2 rounds of running summations/XOR’s separately. In this approach, if 2 messages have identical output from both rounds, then they would be collisions, thus bypassing the post processing made of modular multiplication, bit permutation and AND. While it’s quite feasible to find messages M1 and M2, such that either R1(M1) = R1(M2), or R2(M1) = R2(M2), the outcome of R1 has no bearing on that of R2, and vice versa. Looking for common solutions this way, is therefore effectively no different from a brute force birthday attack. In fact, it can be shown that in the simple case of m = 3 and h = 2, where m is the number of message bytes and h is the number of hash bytes, the solutions for the 2 rounds are mutually exclusive, out of the 256 R1 collisions for a given R1 hash, no pair collide in R2.

### A.9 RSH Differential Cryptanalysis

This is to find m, ∆m with ∆m ≠ 0 and ∆h(m) = 0, where ∆m = m ^ m’ and ∆h(m) = h(m) ^ h(m’), where ^ can be + instead or in conjunction. It is effective in 2 scenarios, one is if h is made of ^ and + only, for obvious reasons, the other is if h is made of mapping, as a simple example, {0, 1, 2, 3} → {0, 3, 2, 1}. In the latter case, if ∆m = m + m’ = 1, then m/m’ can be 0/1 or 2/3 leading to h(m)/h(m’) = 0/3 or 2/1, or ∆h(m) = h(m) + h(m’) = 3, thus distinctively uneven. RSH is beyond these, the inaccuracy of the linear approximation of modular multiplication, bit permutation and AND is no less than the hash size complexity, thus negating the feasibility of this approach.

### A.10 RSH Dry Run

Data: For people still clinging to decent jobs, the challenge is more complicated.

(32 bytes) Hash: 170 115 252 234 103 19 126 229 230 111 89 107 55 248 87 124 164 198 116 144 236 113 106 60 220 140 145 244 203 127 115 77

Now change the last character in the data from period to comma.

Data: For people still clinging to decent jobs, the challenge is more complicated,

(32 bytes) Hash: 93 121 221 107 248 229 184 85 126 97 208 245 203 15 235 65 59 141 210 93 222 52 177 8 215 248 32 13 175 249 6 179

Data: a

(32 bytes) Hash: 55 147 103 139 191 97 126 31 68 96 177 67 208 18 35 119 225 138 26 60 10 7 170 189 20 129 30 165 166 91 43 63

Data: b

(32 bytes) Hash: 128 41 130 165 39 135 222 154 229 250 85 87 13 101 203 153 29 54 34 51 67 166 91 162 85 39 173 56 177 194 33 95

### A.11 RSH burst error correction

If the number of corrupted data bytes is k and k < = n, where n is the number of hash bytes, then the corrupted bytes can be solved from k quasi-linear equations out of the hash. But because any overflow beyond 1 byte is lost from the hash, the solution has some collisions. SoΣ (di x i) is stored in a 2 bytes integer, with i ranging from 1 to m, where m is the total number of data bytes contained in the hash. It is attached to the hash along with 1 byte total sum and 1 byte total XOR. The collisions are matched with the attachment to single out the real solution. Obviously this can only work if the hash is intact. So duplicates of hash may be attached to both ends of the data.

Original data: 89 108 200 234 183 33 85 206 229 5 **[[** 226 164**]]** 59 144 121 215 36 223 48 178 116 147 176 …

Received data: 89 108 200 234 183 33 85 206 229 5 **[[** 221 194**]]** 59 144 121 215 36 223 48 178 116 147 176 …

Corrected data: 89 108 200 234 183 33 85 206 229 5 [[226 164]] 59 144 121 215 36 223 48 178 116 147 176 …
